# Variation in VKORC1 Is Associated with Vascular Dementia

**DOI:** 10.3233/JAD-201256

**Published:** 2021-04-06

**Authors:** Jure Mur, Daniel L. McCartney, Daniel I. Chasman, Peter M. Visscher, Graciela Muniz-Terrera, Simon R. Cox, Tom C. Russ, Riccardo E. Marioni

**Affiliations:** a Lothian Birth Cohorts group, Department of Psychology, University of Edinburgh, Edinburgh, UK; b Centre for Genomic and Experimental Medicine, Institute of Genetics and Molecular Medicine, University of Edinburgh, Edinburgh, UK; c Alzheimer Scotland Dementia Research Centre, University of Edinburgh, Edinburgh, UK; d Division of Preventive Medicine, Brigham and Women’s Hospital & Harvard Medical School, Boston, MA, USA; e Institute for Molecular Bioscience, University of Queensland, Brisbane, QLD, Australia; f Edinburgh Dementia Prevention, University of Edinburgh, Edinburgh, UK; g Division of Psychiatry, Centre for Clinical Brain Science, University of Edinburgh, Edinburgh, UK

**Keywords:** Alzheimer disease, genetics, vascular dementia, warfarin

## Abstract

**Background:**

The genetic variant rs9923231 (*VKORC1*) is associated with differences in the coagulation of blood and consequentially with sensitivity to the drug warfarin. Variation in *VKORC1* has been linked in a gene-based test to dementia/Alzheimer’s disease in the parents of participants, with suggestive evidence for an association for rs9923231 (*p* = 1.8×10^–7^), which was included in the genome-wide significant *KAT8* locus.

**Objective:**

Our study aimed to investigate whether the relationship between rs9923231 and dementia persists only for certain dementia sub-types, and if those taking warfarin are at greater risk.

**Methods:**

We used logistic regression and data from 238,195 participants from UK Biobank to examine the relationship between *VKORC1*, risk of dementia, and the interplay with warfarin use.

**Results:**

Parental history of dementia, *APOE* variant, atrial fibrillation, diabetes, hypertension, and hypercholesterolemia all had strong associations with vascular dementia (*p* < 4.6×10^–6^). The T-allele in rs9923231 was linked to a lower warfarin dose (β_perT - allele_ = –0.29, *p* < 2×10^–16^) and risk of vascular dementia (OR = 1.17, *p* = 0.010), but not other dementia sub-types. However, the risk of vascular dementia was not affected by warfarin use in carriers of the T-allele.

**Conclusion:**

Our study reports for the first time an association between rs9923231 and vascular dementia, but further research is warranted to explore potential mechanisms and specify the relationship between rs9923231 and features of vascular dementia.

## INTRODUCTION

Warfarin is the most prescribed anticoagulant worldwide [1] and is commonly used as a treatment for atrial fibrillation (AF) [2]. The drug functions by inhibiting the enzyme vitamin K epoxide reductase (VKOR), effectively interfering with the vitamin K cycle required for coagulation of blood [3]. As a result of variations in age, height, weight, genotype, and other factors [4–6], patients vary up to 20-fold in their sensitivity to warfarin [7]. Clinically, the optimum dose is estimated using tests of blood coagulation, commonly the International Normalized Ratio (INR). The strongest genetic predictor of warfarin sensitivity is the gene *VKORC1*, which encodes for the vitamin K epoxide reductase subunit 1 (VKORC1) and acco-unts for approximately a third of the variance in warfarin sensitivity [3]. Three *VKORC1* SNPs, rs9923231, rs9934438, and rs2359612—which are in very high linkage disequilibrium—are the best gen-etic predictors of warfarin sensitivity [3, 7, 8].

In a recent genome-wide association study (GWAS) meta-analysis of parental dementia and case-control Alzheimer’s dementia (ADem) [9], *VKORC1* was associated (after Bonferroni correction) with ADem in a gene-based test (*p* = 5.1×10^–8^); the T-allele in rs9923231, which is related to the need for a lower dose of warfarin, was not a genome-wide significant finding, but was both located within a genome-wide significant locus and nominally associated with an increased risk of ADem (*p* = 1.8×10^–7^). Pure Alzheimer’s disease pathology, characterized by amyloid plaques and neurofibrillary tangles in the grey matter, is uncommon, and most patients exhibit a mixed pathology in which vascular factors often play a prominent role [10]. In fact, there is extensive evidence directly linking vascular dysfunction to ADem [11]. Thus, a possible explanation for the findings [9] is that vascular factors played a crucial role in a proportion of the ADem cases/family history cases observed. If that is the case, then there should be an even stronger relationship between *VKORC1* and vascular dementia (VaD) that is mostly due to cardiovascular factors.

Most strokes in western countries are due to occlusions in blood vessels (ischemic), and some are due to ruptures in blood vessels (hemorrhagic) [12]. If carriers of the T-allele in rs9923231 experience a reduction of blood coagulation and subsequent seq-uential minor hemorrhagic strokes, the resulting pa-thology could manifest in dementia and explain the observed link. Furthermore, compared to non-carriers of the T-allele, patients with AF that carry the T-allele could be at an increased risk of intracerebral hemorrhage and consequentially VaD when prescribed warfarin. Here, we study the same UK Biobank cohort as previously [9], but consider both individual and parental dementia status. We test whether T-allele status is associated with an increased risk of VaD and explore whether carriers of the T-allele are at a greater risk of VaD than non-carriers when prescribed warfarin.

## METHODS

### Sample

We used data from UK Biobank, a large and detailed prospective study of over 500,000 participants aged 37–73 that were recruited between the years 2006 and 2010. UK Biobank has been described in detail before [13]. The Research Ethics Committee (REC) granted ethical approval for the study (reference 11/NW/0382) and the current analysis was conducted under data application 10279.

### Genotyping

**Fig. 1:**
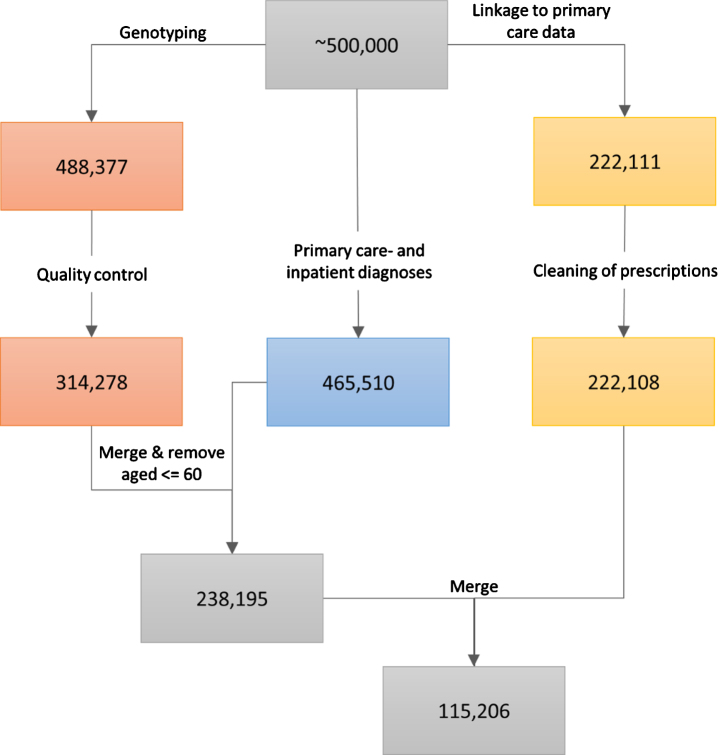
The data cleaning procedure. The left path (orange boxes) represents the genotyping and associated quality control, the middle path (blue box) represents the ascertainment of primary care- and inpatient diagnoses, and the right path (yellow boxes) represents the linkage to primary care prescriptions and the cleaning of the latter. The last two steps (grey boxes) involve the inclusion of only those participants that were older than 60 at the end of sampling and who passed through the left and middle paths (first grey box, 238,195 participants), or through all three data-cleaning paths (second grey box, 115,206 participants). All analyses that did not include prescribing data in the models were performed using the 238,195 participants, while the analyses that utilized warfarin prescription history used the 115,206 participants.

Details on genotyping in the UK Biobank have been reported before [14, 15]. Briefly, for 49,950 participants, genotyping was performed using the UK BiLEVE Axiom Array, and for 438,427 participants, genotyping was performed using the UK Biobank Axiom Array. The released data contained 805,426 markers for 488,377 participants. Further quality control steps were performed as previously reported [9]. They included the removal of outliers, of incongruent data points, and of related participants using a relationship cut-off of 0.025 (GCTA GREML) [16]. This left an unrelated cohort of 314,278 individuals of white British ancestries (Fig. 1).

### Warfarin prescription data

The UK Biobank obtained data on prescriptions for 222,111 participants via primary care computer system suppliers (EMIS Health and Vision for Scotland, and Wales, Vision and The Phoenix Partnership for England) and has engaged other intermediaries (Albasoft, a third-party data processor, for Scotland and the SAIL databank for Wales). All participants provided written consent for linkage to their health records upon recruitment to UK Biobank. The data were extracted in May 2017 for Scotland, in September 2017 for Wales, and in June, in July, and in August 2017 for England. The data include the exact dates of prescriptions, drug codes (BNF, Read v2, CTV3, and dm + d), names of drugs as written on the prescription, and, where available, the dosages of prescribed drugs. Empty prescriptions, prescriptions without a date, and duplicate prescriptions (defined as identical prescriptions issued to the same person on the same day) were removed from the sample. This resulted in the removal of 1,467,547 prescriptions. Three participants were completely removed from the dataset (Fig. 1). Warfarin prescriptions were extracted by searching for the word “warfarin” under the name/content of each prescription. For each participant, we calculated warfarin use by summing the number of days on which warfarin was prescribed, and warfarin dose by averaging the prescribed dose over all prescriptions of warfarin.

### Disease status

Data on diagnoses for 465,510 participants were obtained by the UK Biobank from two sources: 1) from primary care similarly to the prescriptions described above, and 2) from hospital inpatient admissions data. Inpatients are defined as people who are admitted to hospital and occupy a hospital bed. These data included Hospital Episode Statistics for England, Scottish Morbidity Records for Scotland, and the Patient Episode Database for Wales. People with record of any dementia were included in a broad dementia category of “general dementia” that included ADem and VaD, as well as other types of dementia. Furthermore, narrower, more specific categories (ADem, VaD) were also identified. Information on the codes used in the extraction of each diagnosis is provided in Supplementary Table 1. We excluded from our analyses all participants that were 60 years old or younger on the last date of sampling (June 30, 2020) since dementia risk increases steeply with age. Parental diagnoses were ascertained during the initial assessment by asking participants about the presence of “Alzheimer’s disease/dementia” for both mother and father. In our analyses, the parental diagnosis of dementia was considered positive if at least one parent was reported to have suffered from the disorder.

### Models

All analyses where the outcome variable was continuous were performed using linear regression; all models where the outcome variable was binary were performed using logistic regression. All models were controlled for the assessment center in which the par-ticipant was tested, the genotyping- batch and array, 40 genetic principal components, the age, sex, edu-cation, socioeconomic deprivation, alcohol consumption, smoking, physical activity, and body mass index (BMI) of the participants. The models predicting VaD were subsequently additionally controlled for *APOE* variant, concentration of triglycerides (mmol/L), and the diagnoses of hypertension, hypercholesterolemia, and diabetes. All covariates were ascertained immediately prior to or during the participants’ recruitment to the UK Biobank. For education, a binary classification was used that indicated whether a graduate degree had been attained. For socioeconomic deprivation, the Townsend index [17] was used, where higher values indicate greater socioeconomic deprivation (range in the sample: –6.3–10.8). For alcohol consumption, a 6-level scale of frequency of alcohol consumption was used, where 1: “daily or almost daily”, 2: “three or four times a week”, 3:“one or two times a week”, 4: “one to three times a month”, 5: “special occasions only”, 6: “never”. For smoking, the participants were classified as non-smokers, past smokers, or current smokers. For physical activity, the scale provided by the UK Biobank was reduced to a 3-level scale, indicating light, moderate, or strenuous physical activity, as has been used before [18]. For *APOE* genotype based on the nucleotides at SNP positions rs429358 and rs7412, participants with the ɛ3/ɛ3 haplotype were denoted as carrying variant ɛ3, participants with the ɛ2/ɛ2 or ɛ2/ɛ3 haplotypes were denoted as carrying variant ɛ2, and participants with the ɛ3/ɛ4 or ɛ4/ɛ4 haplotypes were denoted as carrying variant ɛ4. Brain imaging data, including the volume of white matter hyperintensities (WMH), were available for 18,251 participants in the sample. For analysis where WMH was modelled as an outcome, WMH was log-transformed and corrected for intracranial volume. For analyses where parental diagnoses were modelled as outcomes, the ages of each parent (current age or age at death) were included in the models. In all cases where we tested for associations between rs9923231 (*VKORC1*) and any form of dementia, we assumed an additive genetic effect for rs99232331. All covariates were simultaneously added to the model and the models were not corrected for multiple comparisons. The effects are reported in odds ratios (OR’s) or unstandardized beta-coefficients. All analyses were performed in R version 3.6.3. The code for preparing and analyzing the data is available at https://github.com/JuM24/VKORC1-and-VaD.

## RESULTS

### Sample characteristics

Among the 238,195 participants, 129,034 (54.2%) were female and 109,161 (45.8%) were male (Table 1). The age range at recruitment was 46–74 years and the median age was 60.9 years (IQR = 9.1). The demographic characteristics of the sub-sample used for analyses utilizing prescription history (Fig. 1) were very similar to the entire sample (Supplementary Table 2). A total of 13,361 (5.6%) participants had been diagnosed with AF and among the 115,206 participants with data on prescriptions, 5,513 (4.8%) had a history of being prescribed warfarin (Supplementary Table 3).

**Table 1:** Demographic characteristics of the sample

Variable	Level	Median (IQR) or *n* (%)			
		All (*n* = 238,195)	General dementia (*n* = 4259)	ADem (1531)	VaD (669)
Age		60.9 (9.1)	65.5 (5.8)	65.9 (5.3)	66.3 (4.7)
Sex	Female	129,034 (54.2)	1,939 (45.5)	795 (51.9)	267 (39.9)
	Male	109,161 (45.8)	2,320 (54.5)	736 (48.1)	402 (60.1)
Education	Graduate degree	72,385 (30.7)	947 (22.6)	313 (20.9)	115 (17.6)
	No graduate degree	163,563 (69.3)	3,235 (77.4)	1,199 (79.1)	539 (82.4)
Deprivation		–2.5 (3.6)	–2.2 (4.2)	–2.3 (4.1)	–2.2 (3.8)
Alcohol consumption	Daily or almost daily	54,261 (22.8)	990 (23.3)	311 (20.3)	152 (22.8)
	3 or 4 times a week	57,255 (24.1)	814 (19.1)	313 (20.5)	117 (17.5)
	1 or 2 times a week	60,106 (25.2)	952 (22.4)	357 (23.3)	147 (22.0)
	1–3 times a month	24,778 (10.4)	396 (9.3)	156 (10.2)	59 (8.8)
	Special occasions only	25,409 (10.7)	576 (13.5)	215 (14.1)	91 (13.6)
	Never	16,239 (6.8)	524 (12.3)	177 (11.6)	101 (15.1)
Smoking	Current smoker	21,470 (9.0)	413 (9.8)	115 (7.6)	74 (11.2)
	Previous smoker	89,608 (37.8)	1,839 (43.4)	647 (42.6)	310 (46.8)
	Non-smoker	126,288 (53.2)	1,983 (46.8)	757 (49.8)	278 (42.0)
Physical activity	Strenuous	19,441 (8.7)	186 (4.9)	74 (5.2)	27 (4.7)
	Moderate	148,812 (66.6)	2,350 (62.1)	908 (64.2)	359 (62.2)
	Light	55,137 (24.7)	1,247 (33.0)	432 (30.6)	191 (28.6)
BMI		26.8 (5.7)	27.1 (6.0)	26.8 (5.6)	27.8 (7.1)
*APOE* variant	ɛ2	30,818 (13.3)	306 (8.2)	79 (5.3)	51 (7.8)
	ɛ3	138,634 (59.7)	1,559 (42.0)	505 (33.6)	275 (42.1)
	ɛ4	62,665 (27.0)	1,846 (49.7)	917 (61.1)	327 (50.1)

**Fig. 2:**
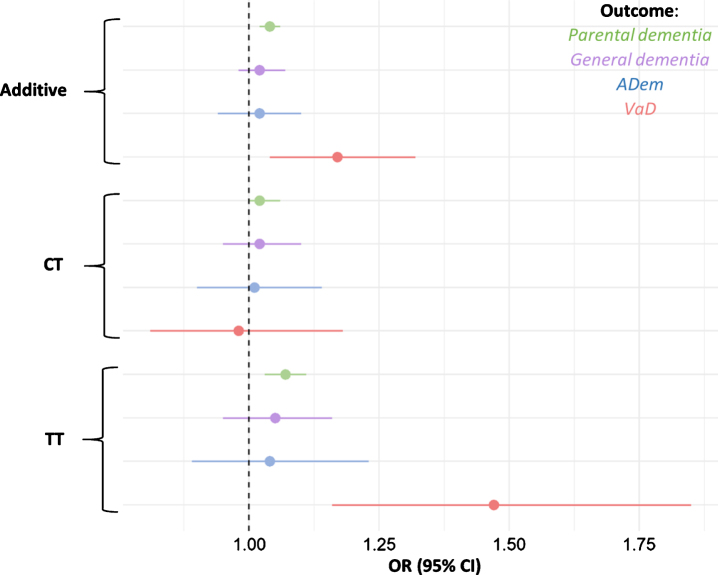
Odds ratios for parental dementia, ADem, and VaD per rs9923231 genotype status. Depicted are the additive effect and the effects of each allele group. The tails represent 95% confidence intervals for the ORs.

There were 145,186 (61.0%) carriers of the T-allele in the sample: 111,756 (46.9%) were heterozygous for the T-allele, and 33,430 (14.0%) were homozygous for the T-allele; the allele frequencies were in Hardy-Weinberg equilibrium (χ*^2^* = 0.23, df = 1, *p* = 0.63). Among the participants, 4,259 (1.8%) had suspected general dementia, 1,531 (0.64%) had suspected ADem (Supplementary Table 4, Supplementary Figure 1), and 669 (0.28%) had suspected VaD (Supplementary Table 4, Supplementary Figure 1); 152 participants (0.03%) had been diagnosed with both ADem and VaD. People with at least one parent with dementia were more likely develop ADem (OR = 3.0, 95% CI = 2.6–3.4, *p* < 2.0×10^–16^) and more likely to develop VaD (OR = 2.1, 95% CI = 1.7–2.7, *p* < 1.9×10^–9^).

### rs9923231 polymorphism and warfarin dose

Carrying the T-allele was negatively associated with the average dose of warfarin (β_perT - allele_ = –0.29, SE = 0.015, *p* < 2.0×10^–16^). Individuals heterozygous for the T-allele were prescribed a dose of war-farin that was on average 0.23 mg smaller than the dose prescribed to non-carriers (SE = 0.022, *p* < 2.0×10^–16^), while individuals homozygous for the T-allele were prescribed a dose of warfarin that was on average 0.62 mg smaller than the dose presc-ribed to non-carriers (SE = 0.032, *p* < 2.0×10^–16^). The average dose of warfarin was also negatively associated with age (β= –0.010, SE = 2.0×10^–3^, *p* = 4.1×10^–7^), and was higher in males (β= 0.062, SE = 0.022, *p* = 5.7×10^–3^).

### rs9923231 polymorphism and dementia risk

Parents of carriers of the T-allele were more li-kely to have developed dementia (additive effect per T-allele: OR = 1.04, 95% CI = 1.02–1.06, *p* = 3.7×10^–5^). When the presence of the T-allele was used to predict general dementia in participants, the effect was not significant, nor was the effect significant when the presence of the T-allele was used to predict ADem in participants (Table 2, Fig. 2).

**Table 2:** Results of the additive models with T as the effect allele, using rs9923231 to predict parental dementia, general dementia, ADem, and VaD

	Effect per T allele			
rs9923231	OR	95% CI	*p*	n cases
Parental dementia	1.04	1.02–1.06	3.7×10^–5^	34,737
General dementia	1.02	0.98–1.07	0.33	4,259
ADem	1.02	0.94–1.10	0.60	1,531
VaD	1.17	1.04–1.32	0.010	669

When limited to the specific outcome of VaD, the additive effect of the T-allele was much larger (OR = 1.17, 95% CI = 1.04–1.32, *p* = 0.010, Table 2, Fig. 2). The full breakdown of all allele groups is shown in Supplementary Table 5. We repeated the models for VaD, with rs9923231 as a predictor and with the simultaneous addition of concentration of triglycerides, *APOE* variant, diagnoses of hypertension (*n* = 84,694), hypercholesterolemia (*n* = 40,363), and diabetes (*n* = 20,990) as additional covariates. While triglycerides, *APOE* variant, hypertension, hypercholesterolemia, and diabetes were significant predictors, this did not affect the relationship between rs9923231 and VaD (Supplementary Table 6). Beca-use of the importance of cardiovascular events in the etiology of VaD, the T-allele was also used to predict stroke, with the full set of covariates as above. The models were not significant for ischemic (*n* = 8,087, OR = 0.98, 95% CI = 0.94–1.01, *p* = 0.21), nor for hemorrhagic (*n* = 2,146, OR = 0.94, 95% CI = 0.88–1.01, *p* = 0.073) stroke. Due to the likely causal link between WMH and dementia [19], rs9923231 was related to WMH in the sample. When all the above covariates were included in the model, the association was significant, with the T-allele negatively associated with WMH (β = –2.3×10^–8^, SE = 7.5×10^–9^, *p* = 2.8×10^–3^).

### Warfarin use and VaD in carriers of the T-allele

In our sample, participants diagnosed with AF were at greater risk for ADem (OR = 1.55, 95% CI = 1.31–1.81, *p* = 1.7×10^–7^) and for VaD (OR = 2.92, 95% CI = 2.38–3.57, *p* < 2.0×10^–16^). The effect remained significant for both ADem (OR = 1.29, 95% CI = 1.08–1.53, *p* = 4.5×10^–3^) and VaD (OR = 2.17, 95% CI = 1.74–2.69, *p* = 1.9×10^–12^) when *APOE* status, triglycerides, and diagnoses of hypercholesterolemia, hypertension, and diabetes were included in the model as covariates. To test whether warfarin use in T-allele carriers diagnosed with AF increases the risk of VaD, we performed a logistic model with AF, warfarin use, and rs9923231 predicting VaD, with the inclusion of a 3-way interaction term between AF, warfarin, and rs9923231. The interaction between AF, warfarin use, and rs9923231 was not significant (OR = 0.99, 95 % CI = 0.98–1.00, *p* = 0.063). The two-way interactions between the above variables were also not significant and effect sizes (main effects) were not substantially attenuated by the addition of the other variables into the models (Supplementary Tables 7and 8). Due to the small number of people with VaD and very limited statistical power for these analyses (Supplementary Text 1), we repeated the analysis by modelling parental dementia as an outcome and including the 3-way interaction term as above; parental dementia was thus treated as a proxy for VaD in the participa-nts. The interaction between AF, warfarin use, and carrier-status was not significant (OR = 0.999, 95% CI = 0.996–1.00, *p* = 0.77).

## DISCUSSION

In this study, we explored the relationship between suspected dementia, atrial fibrillation, warfarin use, and rs9923231, whose T-allele is associated with a reduction in the dose of warfarin [3, 7, 8]. We found a significant association between rs9923231 and suspected VaD, but not between rs9923231 and either suspected general dementia or suspected ADem. While AF was linked to VaD, the use of warfarin in patients that have AF and carry the T-allele did not increase the risk for VaD.

While there have been reports of variants for monogenic forms of VaD [20], data on the genetics of sporadic VaD are sparse. To our knowledge, only two GWAS have been conducted to investigate this: One (*n* = 5,700) [21] found only rs12007229 on the X-chromosome to be linked to incident VaD, while the other (*n* = 284) [22] did not find any significant associations for VaD. A systematic review of all genetic association studies for the broader term of vascular cognitive impairment found an association for 6 SNPs in 6 genes: *APOE*, *ACT*, *ACE*, *MTHFR*, *PON1*, and *PSEN-1*.

Previous research has associated variation in rs9923231 with warfarin dose [3, 7, 8, 24], and with various adiposity-related traits, such as hip circumference, arm- and leg fat mass, and BMI (Gene Atlas [25]). To our knowledge the present study for the first time describes an association between rs9923231 and VaD, although it is important to note that this is not at a genome-wide significant threshold. The lack of a relationship between rs9923231 and either ADem or general dementia in the present study suggests that the association between the T-allele and ADem, as reported previously [9], might have been partly due to the classification of parental dementia. The UK Biobank questionnaire administered to participants did not distinguish between different types of dementia and it is not known how many of the 42,034 parents that were reportedly diagnosed with “Alzheimer’s/dementia” [9] may have suffered from VaD. This hypothesis is further supported by the estimated effect sizes for the association between rs9923231 and ADem, which were not num-erically larger than those for the association between rs9923231 genotype and parental dementia. Since parental dementia was used as a proxy for ADem in participants, the effect for ADem in participants sho-uld have been substantially greater than for parental dementia (even in the absence of statistical significance) if there truly was an association between rs9923231 and ADem (as opposed to an association between rs9923231 and VaD). Furthermore, in a rec-ent GWAS of clinically diagnosed ADem (*n* = 94,437) [26] there was no association between rs9923231 and ADem.

Based on our results and considering the impo-rtance of cardiovascular abnormalities in the pathology of dementia [10, 11], any future studies exploring the association between rs9923231 and dementia must strongly consider the role of cardiovascular factors; The relationship between genotype and dem-entia might hold only for cases of pure VaD or for those in which vascular pathology represents the main cause of the disorder.

There is an established association between dem-entia and both stroke [27] and WMH [28]; thus, stroke or WMH could act as mediators between rs9923231 genotype and VaD. However, we found no evidence for a positive association between either rs9923231 and stroke or rs9923231and WMH. Moreover, the latter association was statistically significant and negative in direction, suggesting participants carrying the T-allele were less likely to exhibit WMH. While we did not directly test for the effects of other relevant processes, including microbleeds and covert stroke, in the relationship between rs9923231 and VaD, given the lack of evidence for an association between rs9923231 and stroke, they are unlikely to act as prominent mediators. These results further complicate the potential relationship between rs9923231 and VaD and reinforce the need for additional studies to confirm this association and to test alternative mechanism distinct from stroke or WMH.

### Interplay between AF, VaD and warfarin use

AF has been previously associated with cognitive decline and dementia. In our study, AF was associated with VaD and with ADem, even after controlling for hypertension and hypercholesterolemia. The association between AF and VaD is unsurprising, considering the inclusion of either vascular disease or history of stroke in almost all definitions of VaD [29]. Despite a substantial overlap of risk factors for AF and ADem, there is some evidence for an independent relationship between the two disorders [30, 31]

Due to the positive association between AF and VaD, the relationship between rs9923231 and VaD, and between rs9923231 and required warfarin dose, T-allele carriers that take warfarin to treat their AF might be at an increased risk of VaD than non-carriers due to warfarin-related brain hemorrhages. To test this, we studied an interaction between warfarin use, AF, and *VKORC1* genotype with VaD. We observed no variation in dementia risk by different combinations of these predictors. Due to reduced coagulation of blood in carriers of the T-allele, these individuals could be at greater risk of internal bleeding when taking warfarin. However, the required dose of warfarin is regularly estimated and adjusted using tests of blood coagulation and based on the results of the present paper, this approach is just as efficient in patients carrying the T-allele.

### Limitations and future directions

The present study has the advantages of having used a well-characterized sample with access to both inpatient- and primary-care diagnoses. However, we acknowledge several limitations. First, despite the large number of people recruited to UK Biobank, the age range at the end of the sampling period for the cohort is 60–83 years, resulting in a low incidence and prevalence of dementia. This heavily reduced the size of our sample, especially when testing for interactions, and led to wide confidence intervals for the estimated odds ratios. Second, despite it not being the only vitamin K antagonist anticoagulant on the UK market, only warfarin was included in the analysis. Third, the dose of warfarin ingested by participants was assumed to correspond to the average of their prescribed dose, despite some individuals possibly taking more or less of the medicine depending on their individual drug regimes. Fourth, clinical diagnoses of dementia subtypes are difficult and are prone to errors due to the presence of comorbidities and cardiovascular factors [32]. In the present paper, imaging data to confirm the diagnoses was unavailable and all diagnoses were based solely on records from primary care and hospitals. Finally, while most definitions of VaD include both dementia and a history of stroke or cardiovascular disease, VaD is very heterogeneous [33]; in the present study, we did not explore potential mechanisms and mediators of the association between rs9923231 and VaD, nor did we test the relationship for different subtypes of VaD.

The knowledge of genetic risk factors for diseases enables the generation of more accurate hypotheses about underlying biological mechanisms and illuminates potential targets for pharmacological intervention. Moreover, it allows for more informed stratification of participants in clinical trials. Studies that build on our research should aim to replicate the findings in a bigger sample and with greater precision determine the effect size for the association between rs9923231 and VaD. Additionally, further work is required to identify possible associations between rs9923231 and features of VaD, such as lacunar infarction, intracerebral hemorrhage, and white matter hyperintensities.

## Supplementary Material

Supplementary Material
