# Supplementary material for: Variation in VKORC1 Is Associated with Vascular Dementia
Source: J Alzheimers Dis. 2021 Apr 6;80(3):1329–37. doi: 10.3233/JAD-201256 (PMC8150662; doi:10.3233/JAD-201256)
Supplement: Supplementary Material [file jad-80-jad201256-s001.pdf]

Supplementary Material

Variation in VKORC1 Is Associated with Vascular Dementia

Supplementary Table 1. Codes used to extract the diagnoses. The columns indicate (1) the disorder of interest, (2) the code corresponding to the disorder, (3) the coding system to which the code belongs, (4) whether the code was part of the inpatient- or the primary-care (GP) record, and (5) how many participants in UK Biobank were assigned a given diagnosis. All codes and coding systems were provided by UK Biobank, which also provides with tables for converting between the different coding systems. Note that any single patient might have been assigned several distinct codes (for the same disorder). AD, Alzheimer's disease/dementia; AF, atrial fibrillation; VaD, vascular dementia

Disorder	Code	Code System	Source	n
AD	XaIkh	Read code	GP	184
AD	Eu00.	Read code	GP	143
AD	Eu002	Read code	GP	46
AD	Eu00z	Read code	GP	16
AD	Fyu30	Read code	GP	1
AD	F110.	Read code	GP	283
AD	F1101	Read code	GP	1
AD	X002x	Read code	GP	32
AD	XaIKB	Read code	GP	24
AD	X0030	Read code	GP	37
AD	X00R2	Read code	GP	25
AD	XaIKC	Read code	GP	6
AD	Y016b	Read code	GP	65
AD	Y016d	Read code	GP	3
AD	E001.	Read code	GP	6
AD	E001z	Read code	GP	3
AD	E000.	Read code	GP	1
AD	E0020	Read code	GP	1
AD	G309	ICD10	Inpatient	1924
AD	F009	ICD10	Inpatient	1348
AD	F002	ICD10	Inpatient	320
AD	G308	ICD10	Inpatient	313
AD	G301	ICD10	Inpatient	81
AD	F001	ICD10	Inpatient	76
AD	G300	ICD10	Inpatient	168
AD	F000	ICD10	Inpatient	101
AD	2901	ICD9	Inpatient	1
AF	G573z	Read code	GP	51
AF	G5732	Read code	GP	1
AF	Xa2E8	Read code	GP	1773
AF	XaOfa	Read code	GP	59
AF	XaOft	Read code	GP	33

AF	X202S	Read code	GP	3
AF	XaeUP	Read code	GP	1
AF	G5730	Read code	GP	4160
AF	G573.	Read code	GP	523
AF	I480	ICD10	Inpatient	5068
AF	I481	ICD10	Inpatient	1018
AF	I482	ICD10	Inpatient	438
AF	I483	ICD10	Inpatient	161
AF	I484	ICD10	Inpatient	72
AF	I489	ICD10	Inpatient	16618
AF	4273	ICD9	Inpatient	65
Diabetes	C10..	Read code	GP	4123
Diabetes	C100.	Read code	GP	41
Diabetes	C1000	Read code	GP	31
Diabetes	XE10E	Read code	GP	21
Diabetes	X40J4	Read code	GP	887
Diabetes	C1001	Read code	GP	109
Diabetes	XE10F	Read code	GP	71
Diabetes	X40J5	Read code	GP	12543
Diabetes	C100z	Read code	GP	8
Diabetes	C101.	Read code	GP	84
Diabetes	C1010	Read code	GP	4
Diabetes	C1011	Read code	GP	3
Diabetes	C101y	Read code	GP	2
Diabetes	C101z	Read code	GP	10
Diabetes	C103.	Read code	GP	1
Diabetes	C1030	Read code	GP	1
Diabetes	C103z	Read code	GP	1
Diabetes	C104.	Read code	GP	3
Diabetes	C1041	Read code	GP	1
Diabetes	C104z	Read code	GP	3
Diabetes	C105.	Read code	GP	30
Diabetes	C1051	Read code	GP	1
Diabetes	C105z	Read code	GP	1
Diabetes	C106.	Read code	GP	22
Diabetes	C1061	Read code	GP	1
Diabetes	C106y	Read code	GP	1
Diabetes	C106z	Read code	GP	4
Diabetes	XE10G	Read code	GP	8
Diabetes	X30Kk	Read code	GP	79
Diabetes	XE10H	Read code	GP	37
Diabetes	X00Ag	Read code	GP	473
Diabetes	XE15k	Read code	GP	38
Diabetes	Xa0lK	Read code	GP	1
Diabetes	XaPmX	Read code	GP	3
Diabetes	XE10I	Read code	GP	5

Diabetes	C1080	Read code	GP	3
Diabetes	C1081	Read code	GP	3
Diabetes	C1083	Read code	GP	1
Diabetes	C1085	Read code	GP	3
Diabetes	C1087	Read code	GP	4
Diabetes	C1088	Read code	GP	16
Diabetes	C1089	Read code	GP	11
Diabetes	C108z	Read code	GP	1
Diabetes	C109.	Read code	GP	5
Diabetes	C1090	Read code	GP	25
Diabetes	C1091	Read code	GP	6
Diabetes	C1092	Read code	GP	3
Diabetes	C1093	Read code	GP	10
Diabetes	C1094	Read code	GP	7
Diabetes	C1095	Read code	GP	3
Diabetes	C1096	Read code	GP	15
Diabetes	C1097	Read code	GP	182
Diabetes	Xa4g7	Read code	GP	10
Diabetes	XaELP	Read code	GP	8
Diabetes	XaEno	Read code	GP	2
Diabetes	XaF04	Read code	GP	6
Diabetes	XaFWG	Read code	GP	3
Diabetes	XaFm8	Read code	GP	1
Diabetes	XaFmL	Read code	GP	1
Diabetes	XaFmM	Read code	GP	1
Diabetes	XaELQ	Read code	GP	190
Diabetes	XaEnp	Read code	GP	2
Diabetes	XaEnq	Read code	GP	5
Diabetes	XaF05	Read code	GP	17
Diabetes	XaFWI	Read code	GP	2
Diabetes	XaFmA	Read code	GP	6
Diabetes	XaFn7	Read code	GP	2
Diabetes	XaFn8	Read code	GP	1
Diabetes	XaFn9	Read code	GP	1
Diabetes	X40J6	Read code	GP	585
Diabetes	C10B0	Read code	GP	5
Diabetes	X40J7	Read code	GP	1
Diabetes	C11y0	Read code	GP	39
Diabetes	X40JI	Read code	GP	1
Diabetes	X40JJ	Read code	GP	6
Diabetes	XaIzM	Read code	GP	9
Diabetes	XaIzN	Read code	GP	7
Diabetes	XaJSr	Read code	GP	2
Diabetes	XaKyW	Read code	GP	2
Diabetes	XaOPu	Read code	GP	2
Diabetes	XaIzQ	Read code	GP	53

Diabetes	XaIzR	Read code	GP	117
Diabetes	XaJQp	Read code	GP	3
Diabetes	XaKyX	Read code	GP	2
Diabetes	X40JB	Read code	GP	9
Diabetes	C10y.	Read code	GP	1
Diabetes	C10z.	Read code	GP	2
Diabetes	X40JA	Read code	GP	3
Diabetes	XaJlR	Read code	GP	1
Diabetes	Xaagd	Read code	GP	8
Diabetes	Xaagf	Read code	GP	7
Diabetes	Cyu20	Read code	GP	2
Diabetes	L1806	Read code	GP	2
Diabetes	2500	ICD9	Inpatient	20
Diabetes	25000	ICD9	Inpatient	40
Diabetes	25001	ICD9	Inpatient	15
Diabetes	25009	ICD9	Inpatient	102
Diabetes	2501	ICD9	Inpatient	3
Diabetes	25010	ICD9	Inpatient	2
Diabetes	25011	ICD9	Inpatient	3
Diabetes	25019	ICD9	Inpatient	9
Diabetes	25029	ICD9	Inpatient	1
Diabetes	2503	ICD9	Inpatient	6
Diabetes	2504	ICD9	Inpatient	16
Diabetes	2505	ICD9	Inpatient	5
Diabetes	2509	ICD9	Inpatient	1
Diabetes	25099	ICD9	Inpatient	6
Diabetes	E100	ICD10	Inpatient	54
Diabetes	E101	ICD10	Inpatient	450
Diabetes	E102	ICD10	Inpatient	157
Diabetes	E103	ICD10	Inpatient	866
Diabetes	E104	ICD10	Inpatient	330
Diabetes	E105	ICD10	Inpatient	164
Diabetes	E106	ICD10	Inpatient	73
Diabetes	E107	ICD10	Inpatient	17
Diabetes	E108	ICD10	Inpatient	56
Diabetes	E109	ICD10	Inpatient	4383
Diabetes	E110	ICD10	Inpatient	136
Diabetes	E111	ICD10	Inpatient	348
Diabetes	E112	ICD10	Inpatient	594
Diabetes	E113	ICD10	Inpatient	3135
Diabetes	E114	ICD10	Inpatient	1230
Diabetes	E115	ICD10	Inpatient	763
Diabetes	E116	ICD10	Inpatient	269
Diabetes	E117	ICD10	Inpatient	15
Diabetes	E118	ICD10	Inpatient	107
Diabetes	E119	ICD10	Inpatient	36924

Diabetes	E121	ICD10	Inpatient	1
Diabetes	E123	ICD10	Inpatient	1
Diabetes	E125	ICD10	Inpatient	1
Diabetes	E128	ICD10	Inpatient	1
Diabetes	E129	ICD10	Inpatient	3
Diabetes	E131	ICD10	Inpatient	10
Diabetes	E132	ICD10	Inpatient	3
Diabetes	E133	ICD10	Inpatient	16
Diabetes	E134	ICD10	Inpatient	3
Diabetes	E135	ICD10	Inpatient	3
Diabetes	E136	ICD10	Inpatient	5
Diabetes	E137	ICD10	Inpatient	1
Diabetes	E138	ICD10	Inpatient	4
Diabetes	E139	ICD10	Inpatient	419
Diabetes	E144	ICD10	Inpatient	57
Diabetes	E145	ICD10	Inpatient	29
Diabetes	E146	ICD10	Inpatient	30
Diabetes	E147	ICD10	Inpatient	3
Diabetes	E148	ICD10	Inpatient	36
Diabetes	O243	ICD10	Inpatient	2
Hypercholesterolemia	C320.	Read code	GP	123
Hypercholesterolemia	XE11S	Read code	GP	6545
Hypercholesterolemia	C3200	Read code	GP	186
Hypercholesterolemia	X40X0	Read code	GP	12
Hypercholesterolemia	C320y	Read code	GP	21
Hypercholesterolemia	C320z	Read code	GP	379
Hypercholesterolemia	Xa9As	Read code	GP	4808
Hypercholesterolemia	2720	ICD9	Inpatient	27
Hypercholesterolemia	27209	ICD9	Inpatient	2
Hypercholesterolemia	E780	ICD10	Inpatient	65264
Hypertension	F282.	Read code	GP	50
Hypertension	G20..	Read code	GP	28
Hypertension	XE0Uc	Read code	GP	30795
Hypertension	XM02V	Read code	GP	2066
Hypertension	XE0Ub	Read code	GP	9795
Hypertension	XaZWm	Read code	GP	314
Hypertension	Xab9L	Read code	GP	143
Hypertension	Xab9M	Read code	GP	14
Hypertension	XaZWn	Read code	GP	1
Hypertension	G2y..	Read code	GP	27
Hypertension	G2z..	Read code	GP	761
Hypertension	XaZbz	Read code	GP	138
Hypertension	3482	ICD9	Inpatient	1
Hypertension	4010	ICD9	Inpatient	2
Hypertension	4011	ICD9	Inpatient	1
Hypertension	4019	ICD9	Inpatient	201

Hypertension	G932	ICD10	Inpatient	163
Hypertension	I10	ICD10	Inpatient	140444
Other dementia	3314	ICD9	Inpatient	5
Other dementia	3318	ICD9	Inpatient	1
Other dementia	F020	ICD10	Inpatient	157
Other dementia	F021	ICD10	Inpatient	1
Other dementia	F022	ICD10	Inpatient	8
Other dementia	F023	ICD10	Inpatient	277
Other dementia	F024	ICD10	Inpatient	1
Other dementia	F028	ICD10	Inpatient	313
Other dementia	F03	ICD10	Inpatient	2737
Other dementia	F09	ICD10	Inpatient	16
Other dementia	G310	ICD10	Inpatient	192
Other dementia	G311	ICD10	Inpatient	5
Other dementia	G312	ICD10	Inpatient	131
Other dementia	G318	ICD10	Inpatient	581
Other dementia	G319	ICD10	Inpatient	1199
Stroke (hem.)	G600.	Read code	GP	6
Stroke (hem.)	G601.	Read code	GP	1
Stroke (hem.)	G60z.	Read code	GP	37
Stroke (hem.)	G61..	Read code	GP	25
Stroke (hem.)	G610.	Read code	GP	4
Stroke (hem.)	G611.	Read code	GP	4
Stroke (hem.)	G612.	Read code	GP	7
Stroke (hem.)	G613.	Read code	GP	14
Stroke (hem.)	G614.	Read code	GP	3
Stroke (hem.)	G617.	Read code	GP	4
Stroke (hem.)	G61z.	Read code	GP	52
Stroke (hem.)	G62..	Read code	GP	2
Stroke (hem.)	G620.	Read code	GP	1
Stroke (hem.)	G621.	Read code	GP	4
Stroke (hem.)	G62z.	Read code	GP	25
Stroke (hem.)	Xa1uW	Read code	GP	416
Stroke (hem.)	Xa01h	Read code	GP	7
Stroke (hem.)	Xa01j	Read code	GP	5
Stroke (hem.)	Xa01k	Read code	GP	4
Stroke (hem.)	Xa01l	Read code	GP	4
Stroke (hem.)	Gyu6E	Read code	GP	3
Stroke (hem.)	XE0VF	Read code	GP	98
Stroke (hem.)	Gyu6F	Read code	GP	2
Stroke (hem.)	XaBM4	Read code	GP	6
Stroke (hem.)	XaBM5	Read code	GP	1
Stroke (hem.)	XE2w4	Read code	GP	18
Stroke (hem.)	XaKK3	Read code	GP	13
Stroke (hem.)	Y00dc	Read code	GP	2
Stroke (hem.)	Y00db	Read code	GP	1

Stroke (hem.)	Gyu60	Read code	GP	1
Stroke (hem.)	Gyu61	Read code	GP	7
Stroke (hem.)	Gyu62	Read code	GP	4
Stroke (hem.)	Gyu6G	Read code	GP	3
Stroke (hem.)	4309	ICD9	Inpatient	30
Stroke (hem.)	4319	ICD9	Inpatient	4
Stroke (hem.)	4320	ICD9	Inpatient	1
Stroke (hem.)	4321	ICD9	Inpatient	1
Stroke (hem.)	4590	ICD9	Inpatient	8
Stroke (hem.)	I600	ICD10	Inpatient	29
Stroke (hem.)	I601	ICD10	Inpatient	153
Stroke (hem.)	I602	ICD10	Inpatient	239
Stroke (hem.)	I603	ICD10	Inpatient	140
Stroke (hem.)	I604	ICD10	Inpatient	46
Stroke (hem.)	I605	ICD10	Inpatient	8
Stroke (hem.)	I606	ICD10	Inpatient	59
Stroke (hem.)	I607	ICD10	Inpatient	51
Stroke (hem.)	I608	ICD10	Inpatient	103
Stroke (hem.)	I609	ICD10	Inpatient	801
Stroke (hem.)	I610	ICD10	Inpatient	170
Stroke (hem.)	I611	ICD10	Inpatient	269
Stroke (hem.)	I612	ICD10	Inpatient	90
Stroke (hem.)	I613	ICD10	Inpatient	54
Stroke (hem.)	I614	ICD10	Inpatient	93
Stroke (hem.)	I615	ICD10	Inpatient	178
Stroke (hem.)	I616	ICD10	Inpatient	52
Stroke (hem.)	I618	ICD10	Inpatient	152
Stroke (hem.)	I619	ICD10	Inpatient	868
Stroke (hem.)	I620	ICD10	Inpatient	658
Stroke (hem.)	I621	ICD10	Inpatient	31
Stroke (hem.)	I629	ICD10	Inpatient	202
Stroke (isch.)	G65..	Read code	GP	30
Stroke (isch.)	XE0VK	Read code	GP	1944
Stroke (isch.)	X00DW	Read code	GP	48
Stroke (isch.)	G65y.	Read code	GP	10
Stroke (isch.)	G65z.	Read code	GP	458
Stroke (isch.)	X00DU	Read code	GP	21
Stroke (isch.)	XE2aB	Read code	GP	707
Stroke (isch.)	G63..	Read code	GP	1
Stroke (isch.)	XE0VG	Read code	GP	4
Stroke (isch.)	XE0VH	Read code	GP	25
Stroke (isch.)	G634.	Read code	GP	168
Stroke (isch.)	X203u	Read code	GP	2
Stroke (isch.)	G63y0	Read code	GP	5
Stroke (isch.)	G63y1	Read code	GP	1
Stroke (isch.)	X00D3	Read code	GP	190

Stroke (isch.)	Xa0kZ	Read code	GP	268
Stroke (isch.)	G640.	Read code	GP	24
Stroke (isch.)	G6400	Read code	GP	9
Stroke (isch.)	G641.	Read code	GP	5
Stroke (isch.)	G6410	Read code	GP	6
Stroke (isch.)	XE0VJ	Read code	GP	135
Stroke (isch.)	X00D9	Read code	GP	1
Stroke (isch.)	Xa00J	Read code	GP	93
Stroke (isch.)	Xa00K	Read code	GP	14
Stroke (isch.)	Y00d0	Read code	GP	2
Stroke (isch.)	G631.	Read code	GP	5
Stroke (isch.)	G64..	Read code	GP	1
Stroke (isch.)	G64z.	Read code	GP	2
Stroke (isch.)	XaBEC	Read code	GP	52
Stroke (isch.)	XaBED	Read code	GP	39
Stroke (isch.)	XaJgQ	Read code	GP	11
Stroke (isch.)	G6760	Read code	GP	4
Stroke (isch.)	G677.	Read code	GP	2
Stroke (isch.)	G6770	Read code	GP	1
Stroke (isch.)	G6771	Read code	GP	1
Stroke (isch.)	G6772	Read code	GP	1
Stroke (isch.)	G683.	Read code	GP	2
Stroke (isch.)	Gyu63	Read code	GP	4
Stroke (isch.)	Gyu64	Read code	GP	9
Stroke (isch.)	4349	ICD9	Inpatient	13
Stroke (isch.)	G458	ICD10	Inpatient	199
Stroke (isch.)	G459	ICD10	Inpatient	3658
Stroke (isch.)	I630	ICD10	Inpatient	69
Stroke (isch.)	I631	ICD10	Inpatient	22
Stroke (isch.)	I632	ICD10	Inpatient	266
Stroke (isch.)	I633	ICD10	Inpatient	563
Stroke (isch.)	I634	ICD10	Inpatient	348
Stroke (isch.)	I635	ICD10	Inpatient	1483
Stroke (isch.)	I636	ICD10	Inpatient	12
Stroke (isch.)	I638	ICD10	Inpatient	433
Stroke (isch.)	I639	ICD10	Inpatient	5088
Stroke (isch.)	I64	ICD10	Inpatient	1657
VaD	XE1Xs	Read code	GP	139
VaD	X003V	Read code	GP	9
VaD	Xa0lH	Read code	GP	8
VaD	X003T	Read code	GP	4
VaD	Eu01z	Read code	GP	12
VaD	Eu01y	Read code	GP	2
VaD	F019	ICD10	Inpatient	1134
VaD	F011	ICD10	Inpatient	41
VaD	F013	ICD10	Inpatient	12

VaD	F010	ICD10	Inpatient	10
VaD	F018	ICD10	Inpatient	11
VaD	F012	ICD10	Inpatient	6

Supplementary Table 2. Demographic characteristics of the sub-sample utilizing prescription history (n=115,206).

Variable	Level	Median (IQR) or n (%)			
		**All** (n=115,206)	**General dementia** (n=1,501)	**ADem** (541)	**VaD** (146)
Age		60.7 (9.2)	64.8 (6.4)	65.2 (5.9)	66.7 (4.9)
Sex	Female	63,001 (52.2)	663 (44.2)	284 (52.5)	58 (39.7)
	Male	52,205 (45.3)	838 (55.8)	257 (47.5)	88 (60.2)
Education	Graduate degree	35,315 (30.9)	359 (24.4)	112 (21.2)	25 (17.6)
	No graduate degree	78,930 (69.1)	1,114 (75.6)	417 (78.8)	117 (82.4)
Deprivation		-2.4 (3.6)	-2.3 (3.9)	-2.3 (3.7)	-1.9 (4.5)
Alcohol consumption	Daily or almost daily	24,481 (22.1)	351 (23.4)	117 (21.6)	30 (20.5)
	3 or 4 times a week	27,875 (24.2)	307 (20.5)	109 (20.1)	20 (13.7)
	1 or 2 times a week	29,607 (25.7)	331 (22.1)	127 (23.5)	38 (26.0)
	1-3 times a month	12,063 (10.5)	133 (8.9)	56 (10.4)	10 (6.8)
	Special occasions only	12,197 (10.6)	191 (12.7)	70 (12.9)	26 (17.8)
	Never	7,909 (6.9)	187 (12.5)	62 (11.5)	22 (15.1)
Smoking	Current smoker	61,723 (9.0)	122 (8.2)	35 (6.5)	16 (11.0)
	Previous smoker	42,741 (37.2)	610 (40.8)	222 (41.2)	67 (45.9)
	Non-smoker	10,361 (53.8)	763 (51.0)	282 (52.3)	63 (43.2)
Physical activity	Strenuous	9,415 (8.7)	79 (5.8)	33 (6.5)	3 (2.5)
	Moderate	71,308 (66.0)	821 (60.5)	316 (62.3)	66 (54.5)
	Light	27,262 (25.2)	457 (33.7)	158 (31.2)	52 (43.0)
BMI		26.9 (5.7)	27.1 (5.7)	26.6 (5.4)	27.0 (6.8)
APOE variant	ε2	14,890 (13.3)	141 (8.4)	42 (5.4)	20 (6.7)
	ε4	67,161 (59.8)	677 (40.3)	270 (34.5)	117 (39.3)
	ε4	30,228 (26.9)	863 (51.3)	470 (60.1)	161 (54.0)

Supplementary Table 3. Frequencies and average ages at recruitment of participants diagnosed with atrial fibrillation (AF) and of participants with a history of warfarin use.

AF diagnosis	Ever prescribed warfarin	
	Yes (median age in y)	No (median age in y)
**Yes**	2,456 (64.9)	3,871 (63.8)
**No**	3,075 (62.8)	105,832 (60.4)

Supplementary Table 4. Frequencies and median ages (in years) at recruitment and at time of diagnosis of participants with different VKORC1-genotypes, and participants diagnosed with general dementia, with ADem, and with VaD.

		General dementia	
		**Yes (age at recruitment; age at diagnosis)**	**No (age at recruitment)**
**Genotype (rs9923231)**	CC	1,649 (65.5; 72.9)	91,360 (60.9)
	CT	1,986 (65.4; 72.9)	109,770 (60.8)
	TT	624 (65.7; 73.0)	32,806 (60.8)
		**ADem**	
		**Yes (age at recruitment; age at diagnosis)**	**No (age at recruitment)**
**Genotype (rs9923231)**	CC	593 (65.9; 73.1)	92,416 (60.9)
	CT	720 (65.8; 73.0)	111,036 (60.9)
	TT	218 (65.9; 72.4)	33,212 (60.9)
		**VaD**	
		**Yes (age at recruitment; age at diagnosis)**	**No (age at recruitment)**
**Genotype (rs9923231)**	CC	255 (66.5; 74.5)	92,754 (60.9)
	CT	286 (66.0; 73.7)	111,470 (60.9)
	TT	128 (66.2; 74.3)	33,302 (60.9)

Supplementary Figure 1. Age distributions of different groups of participants at the end of the sampling period (June 30, 2020).

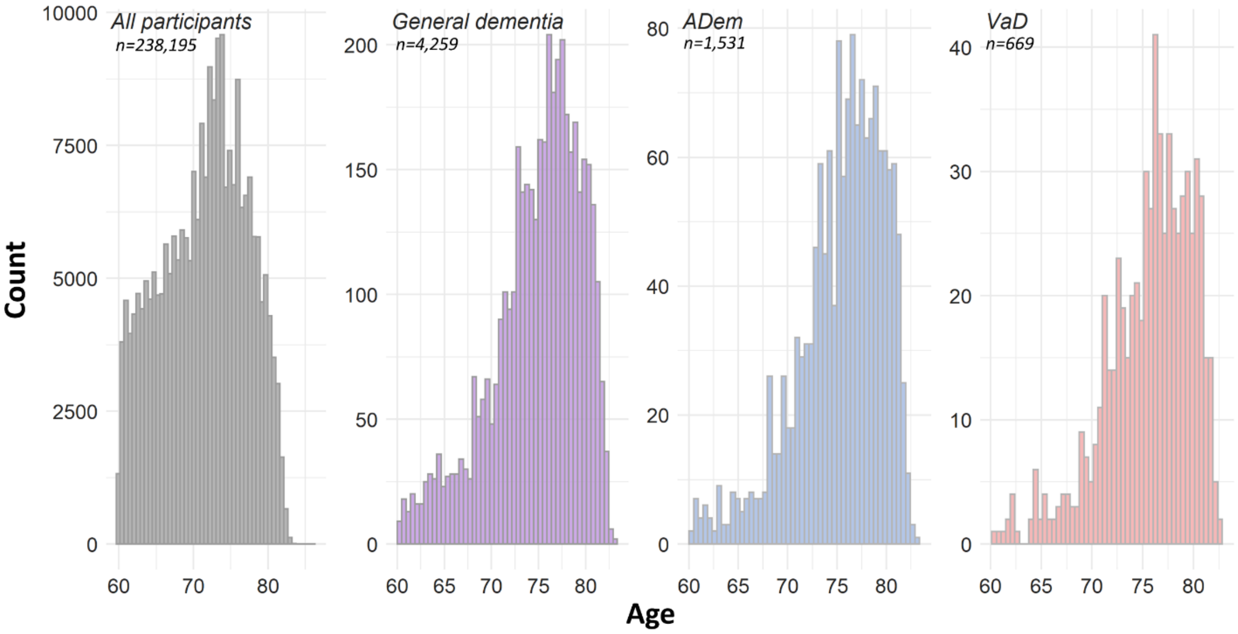

Supplementary Table 5. Logistic regression models with VaD as the outcome and rs9923231 as the predictor. Non-additive model with CC, CT, and TT as rs9923231 genotypes.

rs9923231	OR	95% CI	p	n cases
**Parental dementia**				
CC	1 (ref).			13,235
CT	1.02	1.00-1.06	0.031	16,394
TT	1.07	1.03-1.11	$2.4x10^{-4}$	5,108
**General dementia**				
CC	1 (ref).			1,649
CT	1.02	0.95-1.10	0.56	1,986
TT	1.05	0.95-1.16	0.34	624
**ADem**				
CC	1 (ref.)			593
CT	1.01	0.90-1.14	0.83	720
TT	1.04	0.89-1.23	0.58	218
**VaD**				
CC	1 (ref.)			255
CT	0.98	0.81-1.18	0.84	286
TT	1.47	1.16-1.85	0.0014	128

Supplementary Table 6. Logistic regression models with VaD as the outcome and rs9923231 as the predictor (as an additive effect), with the inclusion of *APOE* status, triglycerides, and diagnoses of hypertension, hypercholesterolemia, and diabetes as covariates. Depicted is the additive effect of the gene, and the effects of selected risk factors. The effect sizes are reported in odds ratios (ORs) or unstandardized beta-coefficients (*).

VaD				
**Variable**	**OR (beta*)**	**95% CI**	**p**	**n cases**
Additive effect	1.20	1.05-1.36	$5.2x10^{-3}$	669
Non-smoker	1 (ref.)			
Previous smoker	1.06	0.87-1.29	0.57	310
Current smoker	1.38	1.00-1.88	0.045	74
BMI	-0.0139*	-0.0141- -0.0137	0.20	
Light physical activity	1 (ref.)			191
Moderate physical activity	0.77	0.63-0.94	$9.3x10^{-3}$	359
Strenuous physical activity	0.82	0.52-1.24	0.37	27
APOE variant ε3	1 (ref.)			276
APOE variant ε2	0.92	0.65-1.28	0.64	51
APOE variant ε4	3.02	2.51-3.64	$4.3x10^{-12}$	327
Hypertension	2.93	2.34-3.69	$<2.0x10^{-16}$	517
Hypercholesterolemia	1.56	1.29-1.89	$4.6x10^{-6}$	298
Triglycerides	-0.15*	-0.06--0.25	0.0022	
Diabetes	2.41	1.95-2.98	$3.6x10^{-16}$	225

Supplementary Table 7. Models with VaD as the outcome and two-way interactions between rs9923231, AF, and warfarin use as predictors. The statistics represent the strength of the interaction between the first two variables with the inclusion of the third as a covariate.

**Interaction**	**Covariate**	**Interaction effect**		
		**Beta**	**SE**	**p**
AF*rs9923231	warfarin use	0.10	0.21	0.62
rs9923231*warfarin use	AF	0.0011	0.0026	0.68
AF*warfarin use	*VKORC1*	-0.0031	0.0036	0.39

Supplementary Table 8. Models predicting VaD (top), ADem (middle), and general dementia (bottom). For each outcome, the effects of three variables—rs9923231, warfarin use, and AF—are shown when each variable is predicting the outcome independently of the other two (univariate; left side), or in addition to the other two (multivariate, right side).

VaD	Univariate			Multivariate		
**Predictors**	**$Beta_{log\text{-}odds}$**	**SE**	**p**	**$Beta_{log\text{-}odds}$**	**SE**	**p**
rs9923231	0.158	0.061	0.010	0.297	0.091	0.0011
warfarin use	0.0051	0.0015	$5.6x10^{-4}$	0.0017	0.0019	0.36
AF	1.07	0.103	$<2.0x10^{-16}$	1.07	0.163	$6.6x10^{-11}$

ADem	Univariate			Multivariate		
**Predictors**	**$Beta_{log\text{-}odds}$**	**SE**	**p**	**$Beta_{log\text{-}odds}$**	**SE**	**p**
rs9923231	0.021	0.034	0.60	0.0030	0.056	0.96
warfarin use	0.0013	0.0015	0.36	$1.1x10^{-4}$	0.0016	0.95
AF	0.436	0.083	$1.7x10^{-7}$	0.306	0.128	0.017

General dementia	Univariate			Multivariate		
**Predictors**	**$Beta_{log\text{-}odds}$**	**SE**	**p**	**$Beta_{log\text{-}odds}$**	**SE**	**p**
rs9923231	0.024	0.025	0.33	0.014	0.035	0.69
warfarin use	0.0026	$7.9x10^{-4}$	0.0012	$2.6x10^{-4}$	$9.3x10^{-4}$	0.78
AF	0.707	0.048	$<2.0x10^{-16}$	0.609	0.073	$<2.0x10^{-16}$

Supplementary Text 1. Power analysis for the interaction effect

A post hoc power analysis using as parameters an alpha error rate of 0.05, a sample size of 115,216, a case rate of VaD of 0.0028, and an effect allele frequency of 0.610 revealed that the power required to detect a two-way interaction effect (OR=1.1) between any two of the studied variables (warfarin use, AF, rs9923231) was approximately 5%. The power analysis was performed using the genpwr library in R.
